# Supplementary material for: The Evolution and Characterization of the RNA Interference Pathways in Lophotrochozoa
Source: Genome Biol Evol. 2024 May 7;16(5):evae098. doi: 10.1093/gbe/evae098 (PMC11114477; doi:10.1093/gbe/evae098)
Supplement: evae098_Supplementary_Data [file evae098_supplementary_data.zip › Supplementary_materials.zip!/selected_miRNAs/NoGe_selected_miRNAs/mirdeep_pdf_files/NMRB01000795.1_Notospermus_geniculatus_strain_Ushimado_scaffold795,_whole_genome_shotgun_sequence_20951.pdf]

Provisional ID	:	NMRB01000795.1_Notospermus_geniculatus_strain_Ushimado_scaffold795,_whole_genome_shotgun_sequence_20951
Score total	:	8366.8
Score for star read(s)	:	3.9
Score for read counts	:	8362.3
Score for mfe	:	1.2
Score for randfold	:	0
Score for cons. seed	:	-0.6
Total read count	:	16414
Mature read count	:	16031
Loop read count	:	0
Star read count	:	383

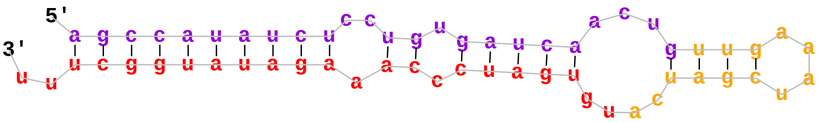

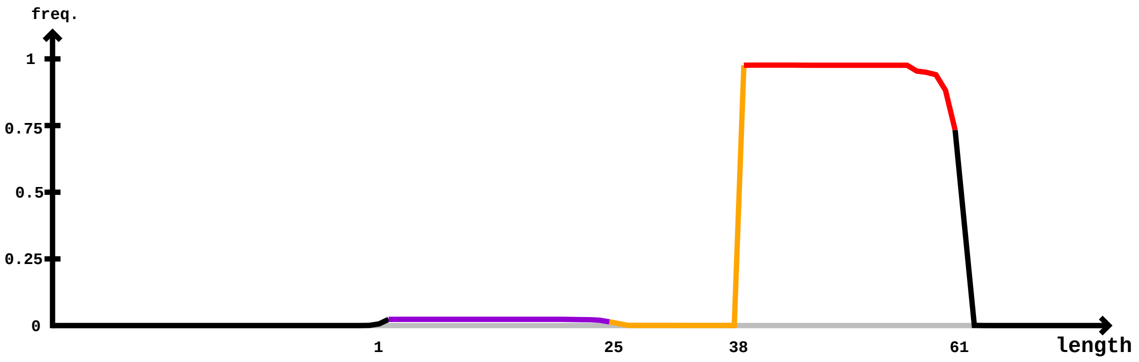


```
                                  Star                                   Mature
5'- accauaaucccugagagaaagaggugacuccacagccauaucuccugugaucaacuguugaaaucgaucaugugaucccaaagauauggcuuuguauguugccucaaa   -3'   obs
    accauaaucccugagagaaagaggugacuccacagccauaucuccugugaucaacuguugaaaucgaucaugugaucccaaagauauggcuuuguauguugccucaaa         exp
    ....................((((((((..((.((((((((((..((.(((((...((((....))))....))))).)).))))))))))).))...))))))))...    reads      mm      sample
    ......................................................................ugugaucccaaagauaugg...................    7          0       Fm2
    ......................................................................ugugaucccaaagauauggA..................    1          1       Fm2
    ......................................................................ugugaucccaaagauauggcu.................    6          0       Fm2
    ......................................................................ugugaucccaaagauauggcG.................    1          1       Fm2
    ......................................................................ugugaucccaaagauauggcuu................    2          0       Fm2
    ......................................................................ugugaucccaaagauaCggcuuu...............    1          1       Fm2
    ......................................................................ugugaucccaaagauauggcuuu...............    20         0       Fm2

    ......................................................................ugugaucccaaagauauggcu.................    2          0       Fm1
    ......................................................................ugugaucccaaagauauggcuu................    3          0       Fm1
    ......................................................................ugugaucccaaagauauggcuuu...............    17         0       Fm1

    ................................cagccauaucuccugugaucaac.....................................................    12         0       Mm1
    ................................cagccauaucuccugugGucaac.....................................................    1          1       Mm1
    ................................cagccauaucuccugugaucaacu....................................................    1          0       Mm1
    .................................agccauaucuccugugaucaacu....................................................    17         0       Mm1
    .................................agccauaucuccugugaucaacug...................................................    7          0       Mm1
    ......................................................................ugugaucccaaagauaug....................    106        0       Mm1
    ......................................................................ugugaucccaaagauauC....................    3          1       Mm1
    ......................................................................ugugaucccaaagauaGg....................    1          1       Mm1
    ......................................................................ugugaucccaaagauauA....................    3          1       Mm1
    ......................................................................ugugaucccaaagauaugg...................    31         0       Mm1
    ......................................................................ugugaucccGaagauaugg...................    1          1       Mm1
    ......................................................................ugugaucccaaagauauCgc..................    1          1       Mm1
    ......................................................................ugugaucccaaagauauggc..................    23         0       Mm1
    ......................................................................ugugaucccaaagauauggA..................    8          1       Mm1
    ......................................................................ugugaucccaaagauauCgcu.................    1          1       Mm1
    ......................................................................ugugaucccaCagauauggcu.................    1          1       Mm1
    ......................................................................ugugaucccaaagauCuggcu.................    1          1       Mm1
    ......................................................................ugugauccUaaagauauggcu.................    1          1       Mm1
    ......................................................................ugugaucccaaagauauggcu.................    171        0       Mm1
    ......................................................................uguCaucccaaagauauggcu.................    1          1       Mm1
    ......................................................................ugugaucccaaagauaAggcu.................    1          1       Mm1
```

```
                                            Star                    Mature
accauaaucccugagagaaagaggugacuccacagccauaucuccugugaucaacuguugaaaucgaucaugugaucccaaagauauggcuuuguauguugccucaaa
......................................................................ugugaucccaaagCuauggcuu................  1    1  Mm1
......................................................................ugugaucccaaagauauAgcuu................  1    1  Mm1
......................................................................ugugGucccaaagauauggcuu................  1    1  Mm1
......................................................................ugugUucccaaagauauggcuu................  1    1  Mm1
......................................................................ugugaucccaaagauauggcuu................  255  0  Mm1
......................................................................Ngugaucccaaagauauggcuu................  1    1  Mm1
......................................................................ugugaucccaaagauauUgcuu................  1    1  Mm1
......................................................................ugugaucccaaagauaCggcuu................  1    1  Mm1
......................................................................ugugaucccaaagauauCgcuu................  1    1  Mm1
......................................................................ugugaucccaaagauaAggcuuu...............  1    1  Mm1
......................................................................ugugaucccaaagauaGggcuuu...............  1    1  Mm1
......................................................................ugugaucccaaagauaCggcuuu...............  2    1  Mm1
......................................................................ugugaucccaGagauauggcuuu...............  2    1  Mm1
......................................................................ugugaucccaaagauauAgcuuu...............  1    1  Mm1
......................................................................ugugaucccaaagauauCgcuuu...............  5    1  Mm1
......................................................................ugugaucccaUagauauggcuuu...............  1    1  Mm1
......................................................................ugugaucccaaagauauggcuuu...............  611  0  Mm1
......................................................................ugugaucccaaagauauggcuuuU..............  19   1  Mm1
.......................................................................gugaucccaaagauauggcuuu...............  1    0  Mm1

...............................acagccauaucuccugugauc........................................................  1    0  Mm2
...............................acagccauaucuccugugauca.......................................................  4    0  Mm2
...............................acagccauaucuccugugaucaa......................................................  1    0  Mm2
...............................acagccauaucuccugugaucaac.....................................................  1    0  Mm2
...............................acagccauaucuccugugaucaacu....................................................  2    0  Mm2
...............................acagccauaucuccugCgaucaacu....................................................  1    1  Mm2
................................cagccauaucuccuguAauc........................................................  1    1  Mm2
................................cagccauaucuccugugauc........................................................  6    0  Mm2
................................cagccauaucuccugugauca.......................................................  7    0  Mm2
................................cagccauaucuccugugaucaa......................................................  2    0  Mm2
................................cagccauaucuccugugaucaac.....................................................  12   0  Mm2
................................cagccauaucuccugugaucaacu....................................................  30   0  Mm2
................................cagccauaucuccugugaucaacG....................................................  1    1  Mm2
................................cagccauaucuccugugaucaacug...................................................  5    0  Mm2
.................................agccauaucuccugugaucaac.....................................................  2    0  Mm2
.................................agccauaucuccugugaucaacu....................................................  35   0  Mm2
.................................agccauaucucUugugaucaacu....................................................  1    1  Mm2
.................................agccauaucuccugugaCcaacug...................................................  1    1  Mm2
.................................agccauaucuccugAgaucaacug...................................................  1    1  Mm2
.................................agccauaucuccugugaAcaacug...................................................  1    1  Mm2
.................................agccauaucuccugugaucaacGg...................................................  2    1  Mm2
.................................agccauaucuccugugaGcaacug...................................................  1    1  Mm2
.................................agccauaucuGcugugaucaacug...................................................  1    1  Mm2
.................................agAcauaucuccugugaucaacug...................................................  1    1  Mm2
.................................agccauaucuccugugaucaacug...................................................  158  0  Mm2
.................................agccauaucuccugugaucaacugu..................................................  1    0  Mm2
.................................agccauaucuccugugaucaacuguugaaUu............................................  2    1  Mm2
..................................gccauaucuccugugaucaacug...................................................  1    0  Mm2
.....................................auaucuccugugaucaacug...................................................  1    0  Mm2
.........................................................uugaaaucgaucaugugau................................  4    0  Mm2
.........................................................uugaaaucgaucaugugauc...............................  2    0  Mm2
.....................................................................Cugugaucccaaagauauggcuuu...............  1    1  Mm2
......................................................................ugugaucccaaagauauA....................  1    1  Mm2
......................................................................ugugaucccaaagauaug....................  168  0  Mm2
......................................................................ugugaucccaaagauauC....................  1    1  Mm2
......................................................................ugAgaucccaaagauaug....................  1    1  Mm2
......................................................................ugugaucccaaagauauU....................  4    1  Mm2
......................................................................ugugaucccaaagauaCgg...................  1    1  Mm2
......................................................................ugugaucccaaagauaugg...................  27   0  Mm2
......................................................................ugugaucccaaagauaugA...................  1    1  Mm2
......................................................................ugugaucccaaagauaugU...................  1    1  Mm2
......................................................................ugugaucccaaagauauggA..................  1    1  Mm2
......................................................................ugugaucccaaagauauUgc..................  1    1  Mm2
......................................................................ugugauccUaaagauauggc..................  1    1  Mm2
......................................................................ugugaucccaaagauauggc..................  80   0  Mm2
......................................................................ugugaucccaaagauCuggcu.................  2    1  Mm2
......................................................................ugugaucccaUagauauggcu.................  1    1  Mm2
......................................................................ugugaucccaaagauauCgcu.................  2    1  Mm2
......................................................................ugugauAccaaagauauggcu.................  1    1  Mm2
......................................................................ugugaucccaaagauauUgcu.................  3    1  Mm2
```

Star Mature

accauaaucccugagagaaagaggugacuccacagccauaucuccugugaucaacuguugaaaucgaucaugugaucccaaagauauggcuuuguauguugccucaaa			
....ugugaucccaaagauaAggcu.................	1	1	Mm2
....ugugaAcccaaagauauggcu.................	2	1	Mm2
....ugugaucccaaagauauggcG.................	7	1	Mm2
....ugugaucccaaagauauggcu.................	514	0	Mm2
....ugugaucccaGagauauggcu.................	2	1	Mm2
....ugugaucccaaagauaCggcu.................	2	1	Mm2
....ugugaucccaaagauaugCcu.................	1	1	Mm2
....uAugaucccaaagauauggcuu................	1	1	Mm2
....ugugaucccaaagauauAgcuu................	7	1	Mm2
....uCugaucccaaagauauggcuu................	2	1	Mm2
....ugugaucccaaCgauauggcuu................	1	1	Mm2
....ugugauccUaaagauauggcuu................	2	1	Mm2
....ugugaucccaaagauaGggcuu................	4	1	Mm2
....Cgugaucccaaagauauggcuu................	1	1	Mm2
....ugugaucAcaaagauauggcuu................	1	1	Mm2
....ugugaucccaaagauauggcuA................	1	1	Mm2
....ugugauccAaaagauauggcuu................	1	1	Mm2
....ugugaucccaaagauauggcuG................	1	1	Mm2
....ugugaucccaaagauauggcuu................	1583	0	Mm2
....uguAaucccaaagauauggcuu................	2	1	Mm2
....ugugaucccaaagauaCggcuu................	2	1	Mm2
....ugugaucccaGagauauggcuu................	2	1	Mm2
....ugugaucccaaagauaugAcuu................	1	1	Mm2
....ugugaucccaaagauauCgcuu................	8	1	Mm2
....ugugaCcccaaagauauggcuu................	1	1	Mm2
....ugugaucccaaagauCuggcuu................	1	1	Mm2
....ugugaucccaaagauauggAuu................	1	1	Mm2
....ugugaucccaaagaGauggcuu................	1	1	Mm2
....ugugaucccaaagauaugUcuu................	2	1	Mm2
....ugugaucccaaagauauggUuu................	1	1	Mm2
....ugugaucccaaagauauUgcuu................	7	1	Mm2
....ugugaucccaaaUauauggcuu................	1	1	Mm2
....ugugaucccaCagauauggcuu................	3	1	Mm2
....ugugaucccaaagauauAgcuuu...............	40	1	Mm2
....ugugaGcccaaagauauggcuuu...............	3	1	Mm2
....ugAgaucccaaagauauggcuuu...............	1	1	Mm2
....ugugaucccaaagauauggcuAu...............	3	1	Mm2
....uguUaucccaaagauauggcuuu...............	1	1	Mm2
....ugugaucccaGagauauggcuuu...............	18	1	Mm2
....ugugaucccaaagauaugUcuuu...............	4	1	Mm2
....ugugaucccaaagCuauggcuuu...............	1	1	Mm2
....ugugaucccaaagauauggcuGu...............	8	1	Mm2
....ugugaucccaaagauUuggcuuu...............	4	1	Mm2
....ugugaucccaaagaGauggcuuu...............	7	1	Mm2
....ugugaNcccaaagauauggcuuu...............	1	1	Mm2
....ugugaucccaaagauauggcGuu...............	2	1	Mm2
....ugugauccUaaagauauggcuuu...............	4	1	Mm2
....uAugaucccaaagauauggcuuu...............	3	1	Mm2
....ugugaucccaaagauauggcAuu...............	5	1	Mm2
....ugugaCcccaaagauauggcuuu...............	1	1	Mm2
....ugugaucccUaagauauggcuuu...............	1	1	Mm2
....ugugaucccaaagauauggcuuC...............	3	1	Mm2
....ugugaAcccaaagauauggcuuu...............	6	1	Mm2
....ugugaucccaaagauauggcuuA...............	6	1	Mm2
....ugugaucAcaaagauauggcuuu...............	1	1	Mm2
....ugugGucccaaagauauggcuuu...............	2	1	Mm2
....ugugaucccaaagauauggcCuu...............	5	1	Mm2
....ugCgaucccaaagauauggcuuu...............	4	1	Mm2
....ugugaucccaaagauaGggcuuu...............	14	1	Mm2
....ugugaucccaaagauGuggcuuu...............	2	1	Mm2
....ugugaucccaaagauCuggcuuu...............	3	1	Mm2
....ugugaucccaaagauauggUuuu...............	3	1	Mm2
....ugugauGccaaagauauggcuuu...............	1	1	Mm2
....ugGgaucccaaagauauggcuuu...............	8	1	Mm2
....ugugaucccaaagauaugNcuuu...............	1	1	Mm2
....ugugaucccaaagauaugCcuuu...............	1	1	Mm2
....uguAaucccaaagauauggcuuu...............	3	1	Mm2
....Agugaucccaaagauauggcuuu...............	2	1	Mm2
....ugugaucccaaCgauauggcuuu...............	1	1	Mm2
....ugugaucccCaagauauggcuuu...............	3	1	Mm2

Star Mature

```
accauaaucccugagagaaagaggugacuccacagccauaucuccugugaucaacuguugaaaucgaucaugugaucccaaagauauggcuuuguauguugccucaaa
......................................................................Ngugaucccaaagauauggcuuu...............  2     1  Mm2
......................................................................ugugauNccaaagauauggcuuu...............  1     1  Mm2
......................................................................ugugaucccaaagauauCgcuuu...............  33    1  Mm2
......................................................................ugugaucccaaagauaAggcuuu...............  11    1  Mm2
......................................................................ugugaucccaaagauauggcuuu...............  8459  0  Mm2
......................................................................ugugaucccaaagauaCggcuuu...............  19    1  Mm2
......................................................................ugugaucccaaagauauggcuCu...............  3     1  Mm2
......................................................................ugugaucccaaagauauUgcuuu...............  23    1  Mm2
......................................................................ugugaucccaUagauauggcuuu...............  9     1  Mm2
......................................................................ugugaucccaaGgauauggcuuu...............  6     1  Mm2
......................................................................ugugaucccaaaUauauggcuuu...............  1     1  Mm2
......................................................................ugugauUccaaagauauggcuuu...............  3     1  Mm2
......................................................................ugugauccAaaagauauggcuuu...............  2     1  Mm2
......................................................................ugugaucccGaagauauggcuuu...............  3     1  Mm2
......................................................................ugugaucccaaagauaugAcuuu...............  1     1  Mm2
......................................................................ugugaucccaaagauauggcuuG...............  7     1  Mm2
......................................................................ugugCucccaaagauauggcuuu...............  1     1  Mm2
......................................................................ugugaucccaaaAauauggcuuu...............  1     1  Mm2
......................................................................ugugaucccaCagauauggcuuu...............  11    1  Mm2
......................................................................ugugaucUcaaagauauggcuuu...............  7     1  Mm2
......................................................................ugugaucccaaagauauggcuuug..............  13    0  Mm2
......................................................................ugugaucccaaagauauCgcuuug..............  1     1  Mm2
......................................................................ugugaucccaaagauauUgcuuug..............  1     1  Mm2
......................................................................ugugaucccaaagauauggcuuuC..............  1     1  Mm2
......................................................................ugugaucccaaagauauggcuuuA..............  17    1  Mm2
......................................................................ugugaucccaaagauauggcuuuU..............  252   1  Mm2
......................................................................ugugaucccaaagauauggcuuuUu.............  5     1  Mm2
......................................................................ugugaucccaaagauauggcuuuguU............  1     1  Mm2
.......................................................................gugaucccaaagauauggcuuu...............  2     0  Mm2
........................................................................ugaucccaaagauauggcuuu...............  1     0  Mm2

................................cagccauaucuccugugaucaa......................................................  2     0  Fm3
.................................agccauaucuccugugaucaacug...................................................  1     0  Fm3
......................................................................ugugaucccaaagauaug....................  1     0  Fm3
......................................................................ugugaucccaaagauauggcuu................  3     0  Fm3
......................................................................ugugaucccaaagauauCgcuu................  1     1  Fm3
......................................................................ugugaucccaaagauauggcuuu...............  7     0  Fm3

................................cagccauaucuccugugauc........................................................  1     0  Mm3
................................cagccauaucuccugugaucaac.....................................................  6     0  Mm3
................................cagccauaucuccugugaucaacu....................................................  5     0  Mm3
.................................agccauaucuccugugaucaacu....................................................  4     0  Mm3
.................................Ngccauaucuccugugaucaacug...................................................  1     1  Mm3
.................................agccauaucuccugugaucaacug...................................................  43    0  Mm3
.................................agccauaucuccugugaucaacuU...................................................  1     1  Mm3
.........................................................uugaaaucgaucaugugauc...............................  1     0  Mm3
......................................................................ugugaucccaaGgauaug....................  1     1  Mm3
......................................................................ugugaucccaaagauaug....................  66    0  Mm3
......................................................................uAugaucccaaagauaug....................  1     1  Mm3
......................................................................ugugaucccCaagauaug....................  1     1  Mm3
......................................................................ugugaucccaaagauauA....................  1     1  Mm3
......................................................................ugugaucccaaagauaugg...................  8     0  Mm3
......................................................................ugugaucccaaagauauggc..................  28    0  Mm3
......................................................................ugugaucccaaagauauggA..................  1     1  Mm3
......................................................................ugugaucccUaagauauggc..................  1     1  Mm3
......................................................................ugugaucccaaagauaGggcu.................  1     1  Mm3
......................................................................ugugaucccaaagauauggcC.................  1     1  Mm3
......................................................................ugugaucccaaagauauUgcu.................  2     1  Mm3
......................................................................ugugaucccaaagauauCgcu.................  1     1  Mm3
......................................................................ugugaucccaaagauaAggcu.................  1     1  Mm3
......................................................................Agugaucccaaagauauggcu.................  1     1  Mm3
......................................................................ugugaucccaaagauauggcG.................  1     1  Mm3
......................................................................ugugaucccaaagauauggcu.................  232   0  Mm3
......................................................................ugugaucccaaagauauAgcuu................  3     1  Mm3
......................................................................ugugaucccaaagauauUgcuu................  1     1  Mm3
......................................................................ugugaucccaaagauauggcGu................  1     1  Mm3
......................................................................ugugaucccaaagauUuggcuu................  1     1  Mm3
......................................................................ugugaucccaaagauaGggcuu................  2     1  Mm3
......................................................................ugugaucccaaagauauggcuG................  2     1  Mm3
......................................................................Ngugaucccaaagauauggcuu................  1     1  Mm3
```

```
                                 Star                                  Mature
accauaaucccugagagaaagaggugacuccacagccauaucuccugugaucaacuguugaaaucgaucaugugaucccaaagauauggcuuuguauguugccucaaa
......................................................................ugugaucccaaagauauCgcuu................   2      1     Mm3
......................................................................uCugaucccaaagauauggcuu................   1      1     Mm3
......................................................................ugugaucccaaagauaAggcuu................   6      1     Mm3
......................................................................ugugaucccaaGgauauggcuu................   2      1     Mm3
......................................................................ugugaucccaUagauauggcuu................   3      1     Mm3
......................................................................ugugaucccaaagauauggcuu................   512    0     Mm3
......................................................................ugugauccUaaagauauggcuuu...............   1      1     Mm3
......................................................................ugugaucccUaagauauggcuuu...............   1      1     Mm3
......................................................................ugugaucccaaagauaugUcuuu...............   1      1     Mm3
......................................................................ugugaucccaUagauauggcuuu...............   3      1     Mm3
......................................................................ugugaucccaaCgauauggcuuu...............   1      1     Mm3
......................................................................ugugaucccaaagauauAgcuuu...............   9      1     Mm3
......................................................................ugugaucccaaagauaGggcuuu...............   8      1     Mm3
......................................................................ugugaucAcaaagauauggcuuu...............   1      1     Mm3
......................................................................uAugaucccaaagauauggcuuu...............   1      1     Mm3
......................................................................ugugaAcccaaagauauggcuuu...............   1      1     Mm3
......................................................................uguCaucccaaagauauggcuuu...............   1      1     Mm3
......................................................................ugugaucccaaagauauUgcuuu...............   3      1     Mm3
......................................................................uguUaucccaaagauauggcuuu...............   1      1     Mm3
......................................................................ugugaucccaaagauaugAcuuu...............   2      1     Mm3
......................................................................ugugaucGcaaagauauggcuuu...............   1      1     Mm3
......................................................................ugugaucccaaagauaCggcuuu...............   5      1     Mm3
......................................................................ugugaucccaaUgauauggcuuu...............   2      1     Mm3
......................................................................ugugaucccaaagaGauggcuuu...............   2      1     Mm3
......................................................................ugugaucccaaagauauggUuuu...............   1      1     Mm3
......................................................................ugugaucccaaagauauggcuuG...............   3      1     Mm3
......................................................................ugugaCcccaaagauauggcuuu...............   1      1     Mm3
......................................................................uUugaucccaaagauauggcuuu...............   1      1     Mm3
......................................................................ugugGucccaaagauauggcuuu...............   1      1     Mm3
......................................................................ugugaucccaaagauauCgcuuu...............   13     1     Mm3
......................................................................ugugaucccaaagauauggcuuA...............   1      1     Mm3
......................................................................ugugaucccaaagGuauggcuuu...............   1      1     Mm3
......................................................................ugugaucccaGagauauggcuuu...............   3      1     Mm3
......................................................................ugugaucccaaagauauggcuuu...............   2121   0     Mm3
......................................................................ugugaucccaaagaAauggcuuu...............   1      1     Mm3
......................................................................ugugaucccaaagauaAggcuuu...............   4      1     Mm3
......................................................................ugugaucccaCagauauggcuuu...............   5      1     Mm3
......................................................................ugugaucccaaagauauggcuuug..............   2      0     Mm3
......................................................................ugugaucccaaagauauggcuuuU..............   67     1     Mm3
......................................................................ugugaucccaaagauauggcuuuA..............   2      1     Mm3
......................................................................ugugaucccaaagauauggcuuuUu.............   2      1     Mm3
.......................................................................gugaucccaaagauauggcuuu...............   2      0     Mm3
```
